# A Triple‐Tunnel Suture‐Bridge Technique for Arthroscopic Fixation of Anterior Cruciate Ligament Tibial Avulsion Fractures

**DOI:** 10.1002/atn2.70188

**Published:** 2026-08-05

**Authors:** Changchao Wang, Liyong Zhang, Shaojian Yin, Yongbin Hu, Yan Huang, Yi xi Meng, Xixi Xu, Yuxin Shi

**Affiliations:** ^1^ Department of Orthopedics and Traumatology Yangzhou Traditional Chinese Medicine Hospital Yangzhou City People's Republic of China; ^2^ Jiangsu Food & Pharmaceutical Science College Huaian China

## Abstract

This paper describes an arthroscopic technique for managing anterior cruciate ligament (ACL) tibial avulsion fractures. The procedure combines a 3‐tunnel anchoring construct with cortical button fixation in a crossed‐loop configuration. The method aims to enhance 3‐dimensional stability and provides improved fixation strength compared with conventional 2‐tunnel approaches. The technique employs standard anterolateral/anteromedial portals with minimal access incisions (<5 mm), which feature 3 precisely placed 2.5 mm bone tunnels (2 anterior and 1 posterior to the anterior cruciate ligament footprint for optimal load distribution). A key point involves placing 1 to 2 cortical buttons at crossed angles, which directly promotes anatomical healing of the fracture site. This technique is particularly advantageous for comminuted fractures involving fragments smaller than 5 mm, offering reduction in soft‐tissue dissection compared with open surgical approaches. The procedure should be performed by senior arthroscopic surgeons with at least 5 years of experience and is contraindicated in patients with osteoporosis (bone density T‐score < −2.5).

## Graphical abstract

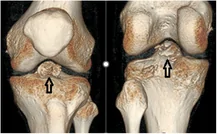

**VIDEO 1:** Injury assessment and preoperative preparation, fracture examination rapidly assesses fracture type closed slash open, evaluates comminution and determines fixation strategy. Hematoma debridement of fracture hematoma while preserving viable tissue bone tunnel preparation anterior cruciate ligament localization. Identify posterior anterior cruciate ligament insertion and drill anatomical tunnels with Kirschner wires (K‐wires). A lumbar puncture needle is used: the needle passes through the bone tunnel, and the guide wire is inserted through the lumbar puncture needle, then pulled in the posterior guiding wire, ensuring the tunnel angle meets biomechanical requirements. Using the same method, position and sequentially pull in the anteromedial and anterolateral guide wires for the anterior cruciate ligament, insert 3 sutures under guidance. Three sutures are passed through the tendon and tied, with both ends of each suture exiting through 2 separate bone tunnels. Additional 2 to 3 non‐knotted sutures may be placed crossing the fracture fragments depending on fracture configuration. Fracture reduction is achieved with traction sutures, which are then tensioned and secured externally with the button plate to provide mechanical stability. Video content can be viewed at https://doi.org/10.1002/atn2.70188.

The management of Meyers‐McKeever type III and IV avulsion fractures of the tibial insertion of the anterior cruciate ligament (ACL) remains a significant challenge. Achieving and maintaining anatomical reduction via a minimally invasive approach is critical for restoring knee stability and function, yet difficult with conventional techniques, especially in comminuted cases. However, conventional fixation methods, such as screws or suture anchors, often prove inadequate for comminuted fractures with small, thin fragments, where secure purchase is difficult to achieve (Figure 1). This technique addresses this clinical challenge by providing a stable suture‐based construct that relies on the robust ACL tissue rather than the size or integrity of the bone fragment for fixation.

**FIGURE 1:**
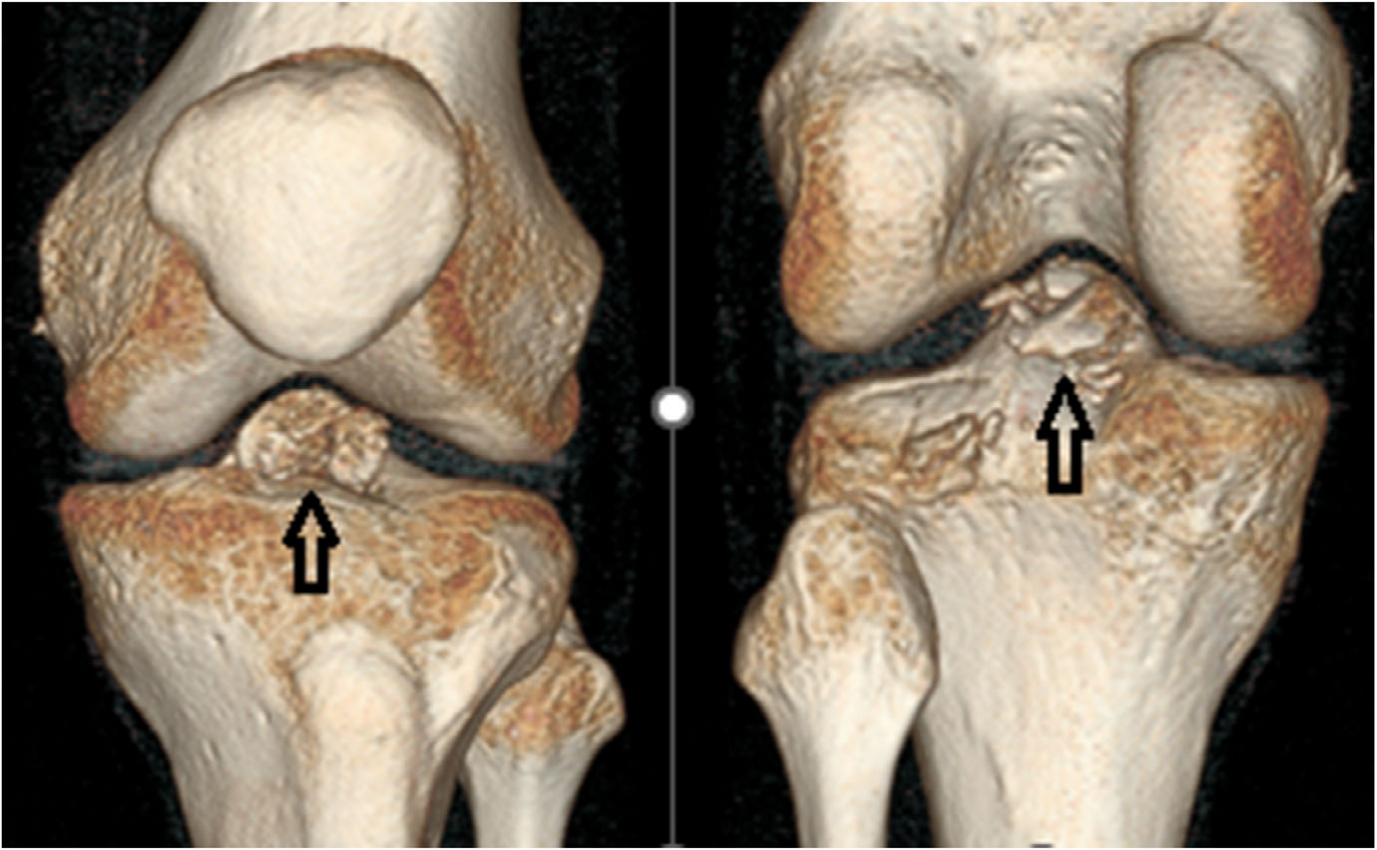
Arthroscopic view of a comminuted avulsion fracture of the ACL tibial insertion (left knee) The image on the left shows the anterior view, and the image on the right shows the posterior view. The arrow indicates the comminuted fragment. (ACL, anterior cruciate ligament.)

## SURGICAL TECHNIQUE

### Patient Positioning and Arthroscopic Examination

After the induction of general anesthesia, the patient is placed in the supine position. A leg holder is used to maintain the affected knee at approximately 90° of flexion. Standard anteromedial and anterolateral arthroscopic portals are established. Diagnostic arthroscopy is performed to evaluate the fracture morphology, and the joint is thoroughly irrigated to evacuate hematoma and any interposed soft‐tissue that may impede reduction.

### Fracture Bed Preparation and Reduction

The fracture bed at the tibial insertion of the ACL is debrided of granulation tissue and fibrous tissue using a shaver and curette to facilitate anatomical reduction of the fragment. Under arthroscopic visualization, the avulsed bone fragment is provisionally reduced and held in its anatomical position using a probe or a Kirschner wire.

### Tibial Tunnel Drilling

**FIGURE 2:**
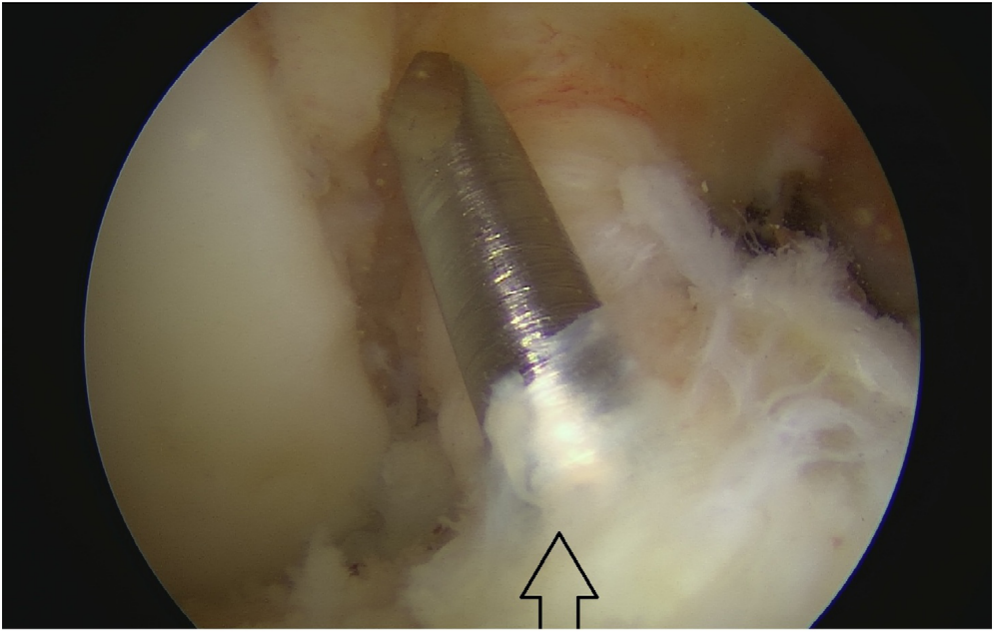
The arrow indicates the anterolateral positioning point of the ACL. (Right knee, anterolateral portal). (ACL, anterior cruciate ligament.)

**FIGURE 3:**
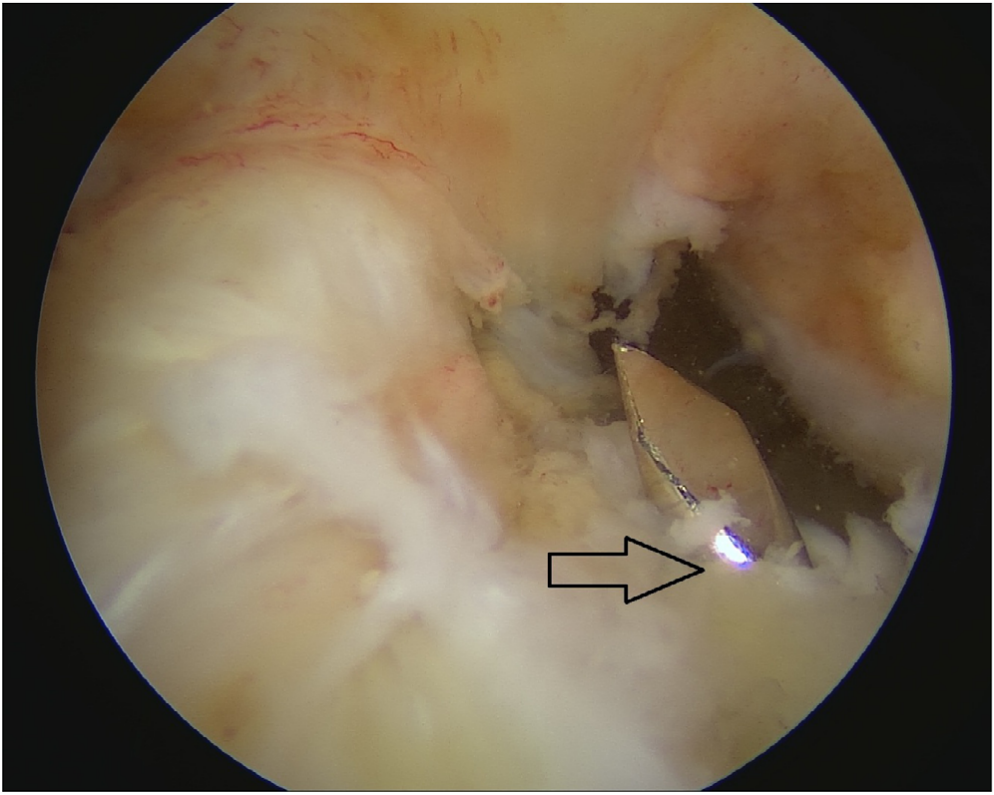
The arrow indicates the anteromedial positioning point of the ACL. (Right knee, anterolateral portal). (ACL, anterior cruciate ligament.)

**FIGURE 4:**
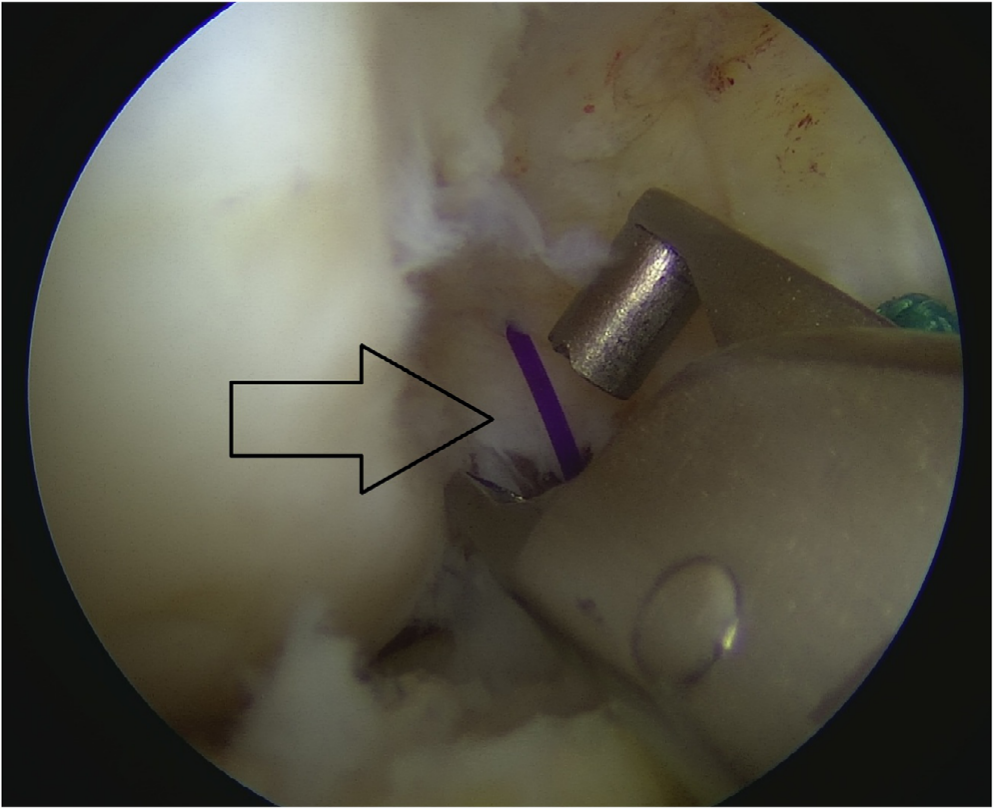
The arrow indicates the anteromedial positioning point of the ACL with a guidewire passed through the bone tunnel for anatomical reconstruction. (Right knee, anterolateral portal). (ACL, anterior cruciate ligament.)

Under arthroscopic and fluoroscopic guidance, 3 bone tunnels (diameter: 2.5 mm) are drilled from the anterior tibial cortex to the margin of the fracture bed. The tunnels are positioned as follows: 2 anterior to the ACL footprint and 1 posterior to it (Figures 2, 3, 4). This triangular configuration is designed to provide optimal biomechanical support.

### Suture Passage and Suture‐Bridge Construction

**FIGURE 5:**
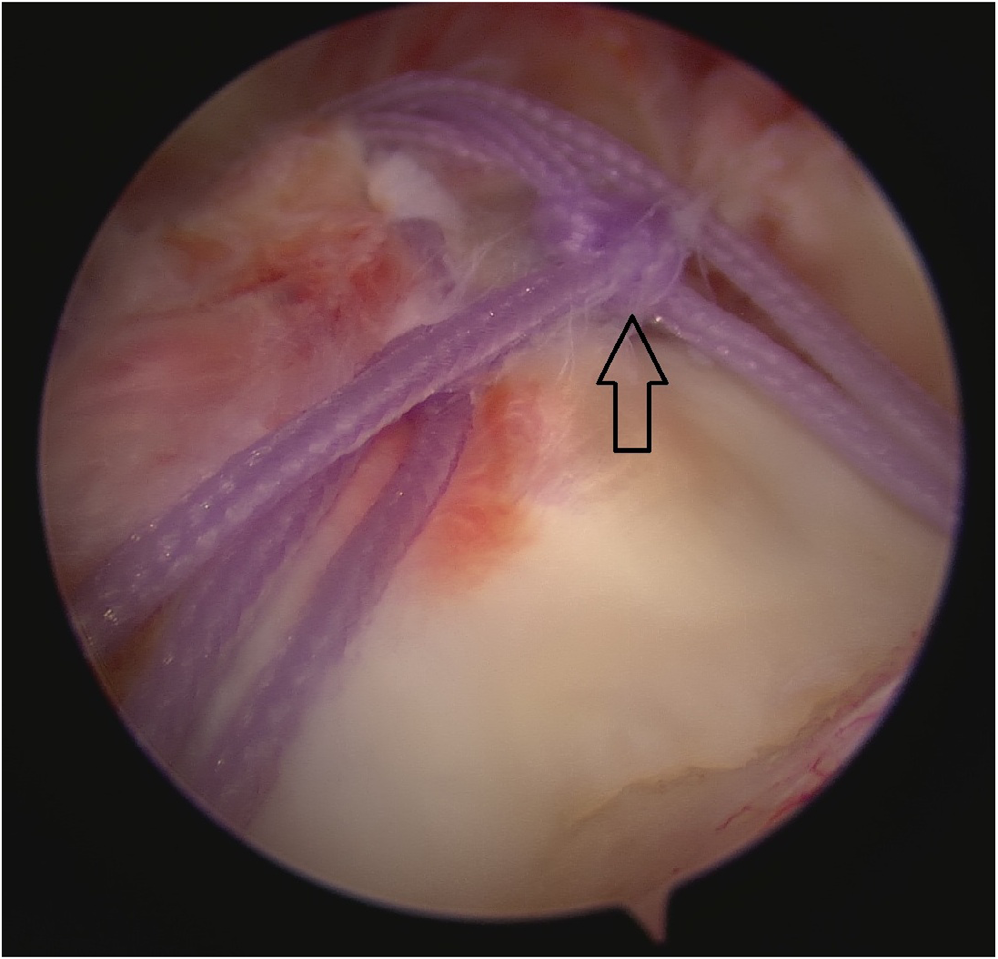
Sutures are passed through the ACL substance and knotted. The arrow indicates the knotted sutures, with each limb exiting through a different tunnel to balance tension. (Right knee, anterolateral portal). (ACL, anterior cruciate ligament.)

**FIGURE 6:**
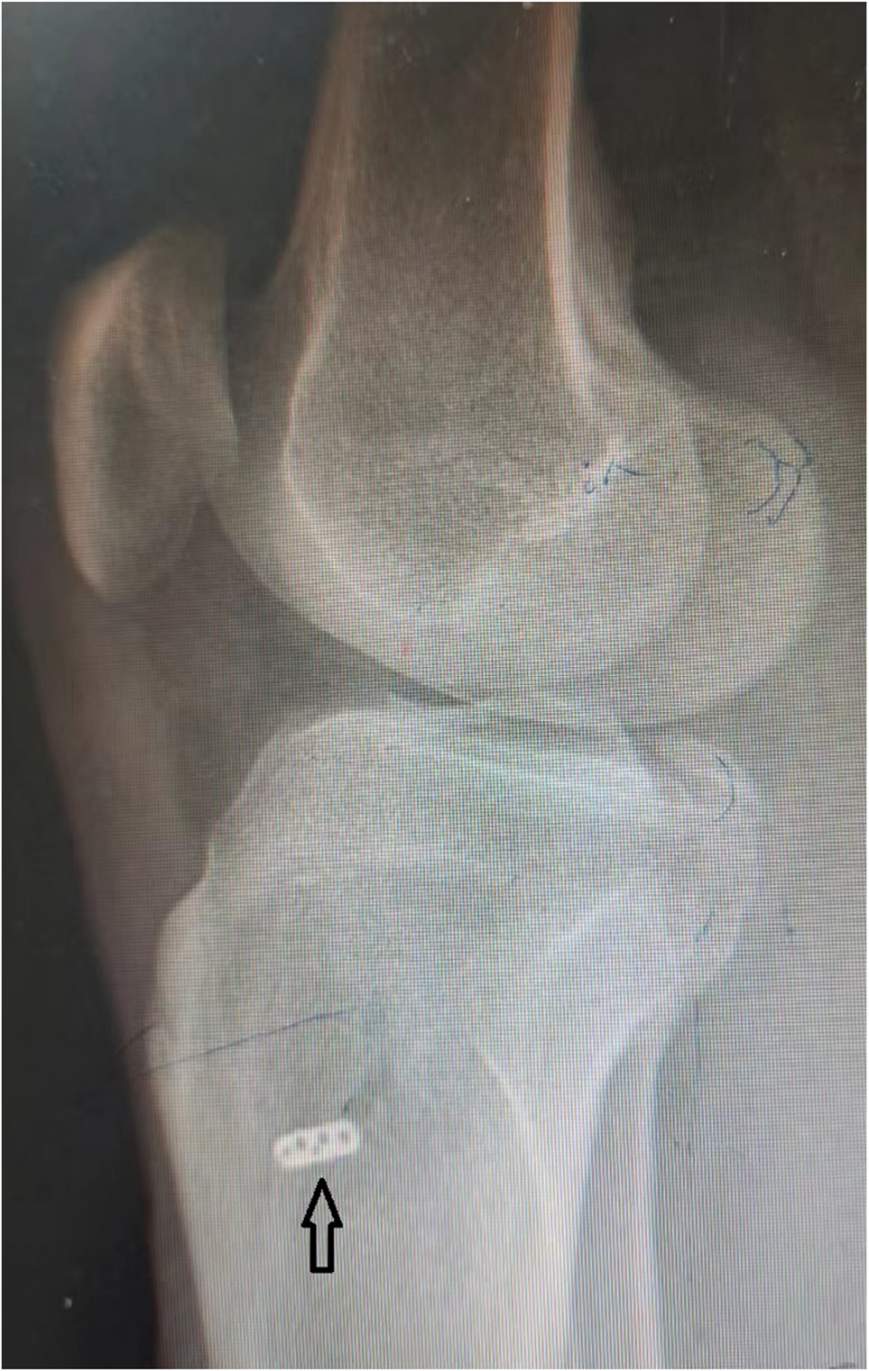
The arrow points to the tendon line fixed with a button plateon the outer side of the tunnel as seen under fluoroscopy, using 1 to 2 button plates as needed. (Right knee, lateral radiograph).

**FIGURE 7:**
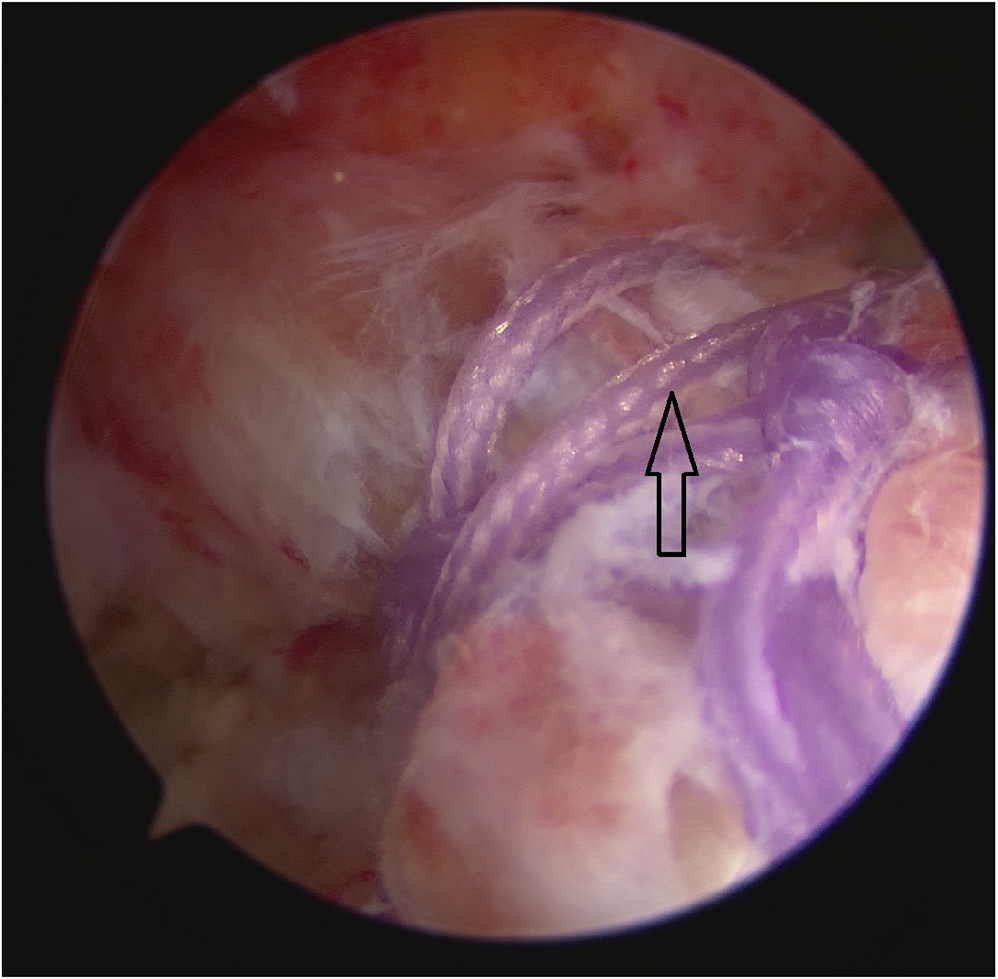
Arrow indicated knotless fixation wire for cross‐bone block compression fixation. (Right knee, anterolateral portal).

**FIGURE 8:**
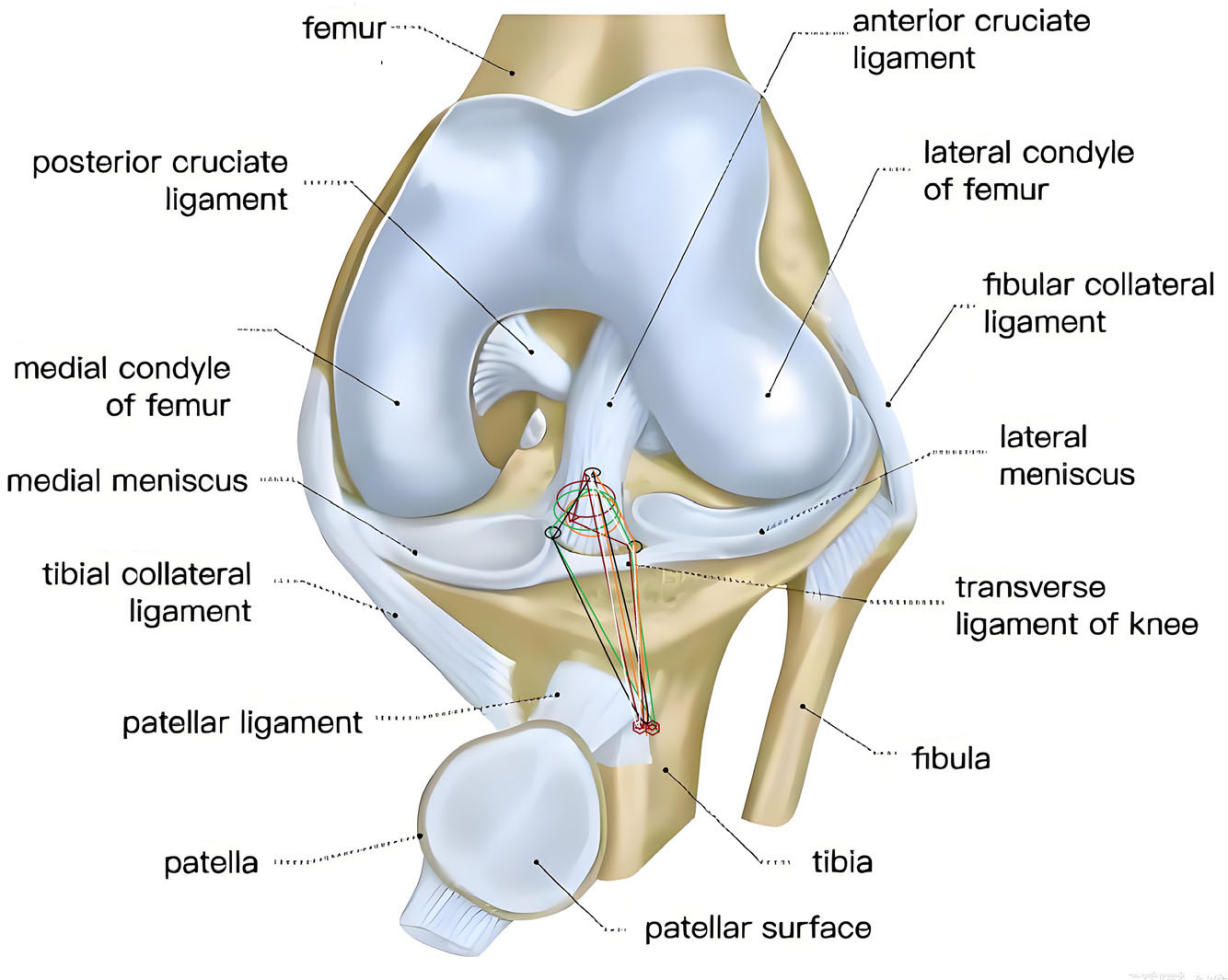
Schematic illustration showing the fixation construct: 3 sutures securing the tendon and 2 to 3 sutures securing the bone fragment.

A suture passer is used to shuttle 6 strands of high‐strength Orthocord sutures (Medos International SARL, Chemin‐Blanc 38, Le Locle, CH‐2400, Switzerland). Three high‐strength sutures through the base of the ACL substance, and each of the 3 sutures is then tied to securely capture the ACL tissue. After knotting, the 2 free limbs of each suture are retrieved through the 2 anterior bone tunnels (Figure 5). Fixation with a RigidLoop button (Medos International SARL, Chemin‐Blanc 38, Le Locle, CH‐2400, Switzerland) outside the tunnel (Figure 6). Three high‐strength sutures fixed the bone. The configuration of the sutures—now encircling the ACL and crossing within the joint—creates a crossed‐loop compression construct designed to compress the fracture fragment (Figure 7) compression structure that encircles the ACL and compresses the fracture fragment. All suture ends are subsequently passed out through the bone tunnels (Figure 8, Video 1).

### Fracture Fixation and Tensioning

With the knee maintained in extension, gentle traction is applied to the proximal tibia to assist reduction. The sutures are then tensioned sequentially. This maneuver reduces the fragment and generates dynamic compression across the fracture site. While maintaining tension, the suture limbs are secured over their respective cortical buttons on the anterior tibial cortex. This final step creates a stable suture‐bridge construct capable of resisting axial and rotational stresses.^1^

Table 1 summarizes the essential technical pearls and corresponding pitfalls for the triple‐tunnel suture‐bridge technique.

**TABLE 1:** Surgical Pearls and Pitfalls

Pearls	Pitfalls
Maintain the knee at 90° of flexion during portal establishment and diagnostic arthroscopy for optimal joint visualization	Inadequate debridement of the fracture bed can impede anatomical reduction
Use a guidewire under fluoroscopic guidance to ensure precise placement of the posterior tunnel relative to the ACL footprint	Drilling the tunnels too close together may compromise the bone bridge strength and lead to convergence
When shuttling sutures, ensure all 6 strands securely capture the robust, proximal ACL substance rather than the fragile avulsed fragment	Over tensioning the sutures with the knee in extension can cause fragment tilting or over‐reduction
Perform sequential tensioning of the suture limbs with gentle anterior tibial traction to achieve dynamic compression across the fracture site	Failing to secure the suture ends over separate cortical buttons may lead to asymmetric compression or construct slippage
Confirm final fixation stability under arthroscopy through a gentle range of motion and probing before closure	In patients with osteoporotic bone (T‐score < −2.5), cortical button purchase may be insufficient, representing a contraindication

ACL, anterior cruciate ligament.

Table 2 outlines the principal advantages and inherent limitations of the triple‐tunnel suture‐bridge technique.

**TABLE 2:** Advantages and Limitations

Advantages	Limitations
Provides a stable, ligament‐based fixation that is independent of small or comminuted fragment size	Requires precise preoperative CT assessment; not suitable for fractures with severely crushed or absent bony attachments
Minimally invasive approach reduces soft‐tissue dissection, morbidity, and risk of infection compared with open techniques	Surgical efficacy is contingent upon surgeon experience in advanced arthroscopy and ligament reconstruction
The triangular tunnel configuration and crossed‐loop suture bridge offer multiplanar stability, resisting axial and rotational stresses	Fixation relies on cortical button purchase on the anterior tibial cortex, which may be compromised in osteoporosis
Utilizes standard arthroscopic instrumentation and implants (e.g., cortical buttons, high‐strength sutures), enhancing reproducibility and cost‐effectiveness	Implant removal (cortical buttons) typically may require a secondary procedure after 12 to 24 months
Enables early postoperative rehabilitation, including prompt isometric exercises and protected range of motion	Indicated for specific fracture patterns (e.g., Meyers‐McKeever type III/IV); not a substitute for all ACL avulsion fracture types

ACL, anterior cruciate ligament; CT, computed tomography.

## DISCUSSION

The development of arthroscopic techniques has markedly advanced the management of ACL avulsion fractures, facilitating a shift from traditional open surgery toward minimally invasive approaches. Current methodologies employ anterograde or retrograde fixation modalities, including cannulated screws, Kirschner wires, and suture anchors, which effectively stabilize large bone fragments.^1^, ^2^ However, these techniques encounter limitations when applied to comminuted fractures characterized by small, thin, or crushed fragments, where direct securement to the bone bed becomes anatomically challenging.^3^ The technique described in this report addresses this specific clinical challenge. It uses a ligament‐based fixation principle, where stability is derived from the strong ACL tissue itself rather than from compromised bone fragments. This approach expands the arthroscopic indications for managing highly comminuted injuries.

The procedural design combines several key features to achieve robust fixation. The triangular configuration of 3 bone tunnels (2 anterior and 1 posterior to the ACL footprint) provides biomechanical support and load distribution. The construction of a crossed‐loop suture bridge, utilizing high‐strength sutures such as Orthocord, encircles the ACL substance and generates dynamic compression across the fracture site. This compression is maintained through suspensory fixation on the anterior tibial cortex using devices such as the RigidLoop Adjustable Cortical Button, creating a stable construct. By employing standard arthroscopic and ligament reconstruction instruments, the procedure shows reproducibility and avoids the need for specialized equipment.

Recent clinical advancements highlight the superiority of arthroscopic‐assisted fixation over conventional anteromedial open reduction, particularly for tibial eminence fractures.^4^, ^5^, ^6^ This technique is suited for comminuted fractures (Meyers‐McKeever type III/IV) with small fragments, where traditional screw or anchor fixation may be inadequate. It mitigates the risks of incomplete reduction associated with open approaches and reduces soft‐tissue dissection.^7^, ^8^ However, its applicability depends on careful patient selection. Preoperative computed tomography imaging is important to evaluate fragment size, displacement, and comminution pattern. The technique is contraindicated in patients with significant osteoporosis (bone density T‐score < −2.5), as fixation relies on cortical button purchase, and its effectiveness may be limited in cases with severely crushed or absent bony ligament attachments.

Postoperatively, a structured rehabilitation protocol is followed to support recovery. A hinged brace is applied immediately, and isometric quadriceps exercises are commenced within 48 hours. Functional exercises are initiated after the fourth week, progressing to partial weight‐bearing at 8 weeks. Full weight‐bearing is permitted only upon radiographic evidence of bone union, typically assessed via X‐ray or computed tomography at 3 to 6 months. The cortical button fixation devices may be electively removed after 12 to 24 months, depending on healing progress.

The decision for surgical intervention is primarily based on the degree of fracture displacement. Minimally displaced fractures may be managed conservatively, whereas significant displacement—particularly when associated with posterior tibial instability—generally necessitates surgical correction to prevent functional deficits.^9^, ^10^ In recent years, even moderately displaced fractures are increasingly treated surgically, as evidence suggests accelerated recovery and improved outcomes compared with nonoperative management.^10^ Historically, open reduction with extensive medial exposure was considered the standard approach for significantly displaced fractures. Although effective, this method was associated with considerable soft‐tissue morbidity.^11^, ^12^ Advances in arthroscopic instrumentation have enabled less invasive techniques, which now allow for anatomical reduction and stable fixation while minimizing soft‐tissue disruption. Various arthroscopic methods have been reported, including fixation with cannulated screws, Kirschner wires, or resorbable implants, particularly in cases with larger fragments amenable to anterograde or retrograde screw or button plate fixation.^13^, ^14^, ^15^, ^16^ Suture anchors provide an alternative reduction mechanism,^17^ though their efficacy diminishes with smaller fragment size.^18^, ^19^ In any arthroscopic pullout technique, accurate visualization of the fracture bed and fragment is crucial,^20^ as is the ability to pass fixation sutures from posterior to anterior across the fragment.^21^ The technique described in this paper employs a suspensory cortical button fixation construct accessed only through standard anteromedial and anterolateral portals, which simplifies surgical workflow.^22^, ^23^ It utilizes 3 bone tunnels and high‐strength sutures to create a crossed‐loop suture bridge, enhancing fixation stability without requiring specialized instruments. This method aims to combine minimal invasiveness with biomechanical reliability, offering a reproducible option that aligns with contemporary surgical principles.

In conclusion, this arthroscopic triple‐tunnel suture‐bridge technique describes a treatment option for comminuted ACL tibial avulsion fractures with small fragments. It provides a ligament‐based fixation strategy in situations where conventional methods are less effective. Future research may focus on comparative clinical outcomes and the integration of technologies such as navigation to further assist in the management of these injuries.

## DISCLOSURES

The authors (C.W., L.Z., S.Y., Y.H., Y.H., Y.M., X.X., Y.S.) declare that they have no known competing financial interests or personal relationships that could have appeared to influence the work reported in this paper.

## FUNDING

Yangzhou Research Program (Collaborative Special Project) in Health and Wellness (2024‐4‐14) Huai'an Municipal Health Commission Research General Project Number (HAWJ202124), The Natural Science Foundation of Huai'an Science and Technology Bureau (HAB202367).

## References

[1] Niu HM , Wang QC , Sun RZ . Therapeutic effect of two methods on avulsion fracture of tibial insertion of anterior cruciate ligament. World J Clin Cases. 2022;10:9641‐9649. 10.12998/wjcc.v10.i27.9641 PMC9516907 36186203

[2] Yu D , Yu R , Zhang J , Chen T , Zhang B. Arthroscopic treatment of adult displaced tibial eminence fractures with anchor and pushlock fixation. Medicine (Baltimore). 2020;99:e21237. 10.1097/MD.0000000000021237 PMC7505351 32957304

[3] Karslioglu B , Guler Y , Dedeoglu SS , Imren Y , Tekin AC , Adas M . Is arthroscopic assisted double tibial tunnel fixation a good option for tibial eminentia fractures. Acta Orthop Belg. 2023;89:117‐121. 10.52628/89.1.10753 37294994

[4] Salvato D , Green DW , Accadbled F , Tuca M . Tibial spine fractures: State of the art. J ISAKOS. 2023;8:404‐411. 10.1016/j.jisako.2023.06.001 37321295

[5] Limone B , Zambianchi F , Cacciola G , Seracchioli S , Catani F , Tarallo L . Management and outcomes of tibial eminence fractures in the pediatric population: A systematic review. Children (Basel). 2023;10:1379. 10.3390/children10081379 PMC10453829 37628378

[6] Johnstone TM , Baird DW Jr , Cuellar‐Montes A , et al. Screws or sutures? A pediatric cadaveric study of tibial spine fracture repairs. Am J Sports Med. 2023;51:2589‐2595. 10.1177/03635465231181059 37382335

[7] Compagnoni R , Maione A , Calanna F , Ferrua P , Randelli PS . Arthroscopic‐assisted reduction and fixation of proximal tibial fractures: Personal surgical technique. Acta Biomed. 2021;92:e2021066. 10.23750/abm.v92i2.10711 PMC8182610 33988160

[8] Hoffmann C , Friederichs J , von Rüden C , Schaller C , Bühren V , Moessmer C . Primary single suture anchor re‐fixation of anterior cruciate ligament proximal avulsion tears leads to good functional mid‐term results: A preliminary study in 12 patients. J Orthop Surg Res. 2017;12:171. 10.1186/s13018-017-0678-9 PMC5683531 29132386

[9] Egger AC , Parikh SN , Wilson PL , et al. What's new in the management of pediatric anterior cruciate ligament tears and tibial spine fractures. Instr Course Lect. 2021;70:399‐414. 33438924

[10] Bomar JD , Edmonds EW . Surgical reduction and fixation of tibial spine fractures in children: Arthroscopic suture fixation. JBJS Essent Surg Tech. 2016;6:e17. 10.2106/JBJS.ST.15.00053 PMC6145633 30237926

[11] Jang KM , Bae JH , Kim JG , Wang JH . Novel arthroscopic fixation method for anterior cruciate ligament tibial avulsion fracture with accompanying detachment of the anterior horn of the lateral meniscus: Three‐point suture fixation. Injury. 2013;44:1028‐32. 10.1016/j.injury.2012.12.008 23312375

[12] Espejo‐Reina A , Prado‐Novoa M , Espejo‐Baena A , Estebanez B , Perez‐Blanca A . Improved tibiofemoral contact restoration after transtibial reinsertion of the anterior root of the lateral meniscus compared to in situ repair: A biomechanical study. Int Orthop. 2023;47:2419‐2427. 10.1007/s00264-023-05769-y PMC10522501 36944816

[13] Wiktor Ł , Tomaszewski R . Results of anterior cruciate ligament avulsion fracture by treatment using bioabsorbable nails in children and adolescents. Children (Basel). 2022;9:1897. 10.3390/children9121897 PMC9776932 36553339

[14] Mahar AT , Duncan D , Oka R , Lowry A , Gillingham B , Chambers H . Biomechanical comparison of four different fixation techniques for pediatric tibial eminence avulsion fractures. J Pediatr Orthop. 2008;28:159‐62. 10.1097/BPO.0b013e318164ee43 18388708

[15] Leie M , Heath E , Shumborski S , Salmon L , Roe J , Pinczewski L . Midterm outcomes of arthroscopic reduction and internal fixation of anterior cruciate ligament tibial eminence avulsion fractures with k‐wire fixation. Arthroscopy. 2019;35:1533‐1544. 10.1016/j.arthro.2018.11.066 30979622

[16] Xu X , Wang H , Cui F , Guo F . Clinical effect of day case arthroscopic surgery in tibial‐eminence fracture in adults using button plates. Front Surg. 2022;9:899438. 10.3389/fsurg.2022.899438 PMC9559727 36248368

[17] Yao J , Wang H , Quan S , Feng W , Cai L , Yang M . Suture‐bridge fixation under arthroscopy in treatment of tibial eminence avulsion fracture of anterior cruciate ligament in adolescents. Chin J Reparative Reconstr Surg. 2018;32:1402‐1405. 10.7507/1002-1892.201804002 PMC8414113 30215491

[18] Kelly S , DeFroda S , Nuelle CW . Arthroscopic assisted anterior cruciate ligament tibial spine avulsion reduction and cortical button fixation. Arthrosc Tech. 2023;12:e1033‐e1038. 10.1016/j.eats.2023.02.052 PMC10390881 37533906

[19] Forkel P , Imhoff AB , Achtnich A , Willinger L . All‐arthroscopic fixation of tibial posterior cruciate ligament avulsion fractures with a suture‐button technique. Oper Orthop Traumatol. 2020;32:236‐247. 10.1007/s00064-019-00626-x 31492968

[20] Willinger L , Imhoff AB , Schmitt A , Forkel P . Fixation of bony avulsions of the posterior cruciate ligament by a suture‐bridge technique. Oper Orthop Traumatol. 2019;31:3‐11. 10.1007/s00064-018-0582-4 30564842

[21] Mutchamee S , Ganokroj P . Arthroscopic transosseous suture‐bridge fixation for anterior cruciate ligament tibial avulsion fractures. Arthrosc Tech. 2020;9:e1607‐e1611. 10.1016/j.eats.2020.05.005 PMC7586735 33134068

[22] Dimitriou D , Zou D , Wang Z , Tsai TY , Helmy N . Anterior root of lateral meniscus and medial tibial spine are reliable intraoperative landmarks for the tibial footprint of anterior cruciate ligament. Knee Surg Sports Traumatol Arthrosc. 2021;29:806‐813. 10.1007/s00167-020-06018-0 32419045

[23] Zhang L , Li C , Zhang J , et al. Significant race and gender differences in anterior cruciate ligament tibial footprint location: A 3D‐based analysis. J Orthop Traumatol. 2023;24:33. 10.1186/s10195-023-00710-w PMC10313598 37389687

